# PIK3R3 is upregulated in liver cancer and activates Akt signaling to control cancer growth by regulation of CDKN1C and SMC1A

**DOI:** 10.1002/cam4.6068

**Published:** 2023-05-22

**Authors:** Weidong Lin, Kunpeng Wang, Jinggang Mo, Liezhi Wang, Zhenshun Song, Hao Jiang, Cong Wang, Chong Jin

**Affiliations:** ^1^ Department of Hepatobiliary Surgery, Shanghai Tenth People's Hospital, School of Medicine Tongji University Shanghai China; ^2^ Department of General Surgery Taizhou Central Hospital (Taizhou University, Hospital) Taizhou China; ^3^ Department of General Surgery The Second Xiangya Hospital, Central South University Changsha China

**Keywords:** cancer growth, CDKN1C, liver cancer, PIK3R3, SMC1A

## Abstract

**Background:**

Liver cancer is a highly malignant disease and the third leading cause of cancer death worldwide. Abnormal activation of PI3K/Akt signaling is common in cancer, but whether phosphoinositide‐3‐kinase regulatory subunit 3 (PIK3R3) plays a role in liver cancer is largely unexplored.

**Methods:**

We determined the expression of PIK3R3 in liver cancer by using TCGA data and our clinical samples and knocked it down by siRNA or overexpressing it by the lentivirus vector system. We also investigated the function of PIK3R3 by colony formation, 5‐Ethynyl‐2‐Deoxyuridine, flow cytometry assay, and subcutaneous xenograft model. The downstream of PIK3R3 was explored by RNA sequence and rescue assays.

**Results:**

We found that PIK3R3 was significantly upregulated in liver cancer and correlated with prognosis. PIK3R3 promoted liver cancer growth in vitro and in vivo by controlling cell proliferation and cell cycle. RNA sequence revealed that hundreds of genes were dysregulated upon PIK3R3 knockdown in liver cancer cells. CDKN1C, a cyclin‐dependent kinase inhibitor, was significantly upregulated by PIK3R3 knockdown, and CDKN1C siRNA rescued the impaired tumor cell growth. SMC1A was partially responsible for PIK3R3 regulated function, and SMC1A overexpression rescued the impaired tumor cell growth in liver cancer cells. Immunoprecipitation demonstrated there is indirect interaction between PIK3R3 and CNKN1C or SMC1A. Importantly, we verified that PIK3R3‐activated Akt signaling determined the expression of CDKN1C and SMC1A, two downstream of PIK3R3 in liver cancer cells.

**Conclusion:**

PIK3R3 is upregulated in liver cancer and activates Akt signaling to control cancer growth by regulation of CDNK1C and SMC1A. Targeting PIK3R3 could be a promising treatment strategy for liver cancer that deserves further investigation.

## Abstract 2

PIK3R3 is upregulated in liver cancer and activates Akt signaling to control cancer growth by regulation of CDNK1C and SMC1A.Targeting PIK3R3 could be a promising treatment strategy for liver cancer that deserves further investigation.

## 1 INTRODUCTION

Liver cancer is the third leading cause of cancer deaths worldwide, with a growing incidence.^1^, ^2^ Hepatocellular carcinoma (HCC) is the most common type of liver cancer, accounting for 90% of cases.^3^ The pathophysiology of HCC is very complex and covers multiple stages including initiation, development, and progression.^4^ Oncogenic drivers and signaling pathways play important roles in each stage. Moreover, HCC can be classified by major molecular drivers and pathways, which are associated with specific genomic disorders, histopathological fingerprints, and clinical outcomes.^5^, ^6^ The main signaling pathways that have been demonstrated include PI3K/Akt/mTOR signaling, Wnt/TGF‐β signaling, Ras/MAPK signaling, and MET and IGF signaling.^7^, ^8^, ^9^

The PI3K signaling pathway is vital for growth, differentiation, and survival. Recent research has uncovered isoform‐specific regulation in class IA and IB PI3Ks^10^ with regulatory subunits playing a crucial role. For example, p85α inhibits p110α activity, while p85β enhances it.^11^ Furthermore, the p110α subunit interacts with other proteins to regulate cellular processes beyond PI3K signaling.^12^ These advancements provide valuable insights into the regulation of the pathway and its impact on cell biology and disease. While the PI3K/Akt signaling pathway is overactivated in different cancers and has been extensively studied in HCC. Abnormal activation of the PI3K/Akt signaling pathway is usually due to mutations in the activation of upstream and downstream Akt effector molecules.

Phosphoinositide‐3‐kinase regulatory subunit 3 (PIK3R3) also called p55PIK, a part of the PI3K regulatory domain, is upregulated in various cancers and plays an important role in tumorigenesis, cell proliferation, and metastasis.^13^ PIK3R3 has been confirmed to be a member of the PI3K/Akt/mTOR pathway by activating it. By targeting PI3K, miR‐29a‐3p modulates PI3K/Akt signaling and hepatic stellate cell proliferation.^14^ Several studies explored the upstream molecules of PIK3R3, especially in a series of microRNAs, and the roles of PIK3R3 in cancers.^13^, ^15^, ^16^, ^17^, ^18^, ^19^, ^20^, ^21^ In HCC, PIK3R3 is overexpressed and could mediate cell proliferation, malignant progression, autophagy, and drug resistance through different upstream molecules, including microRNAs and EVA1.^22^, ^23^, ^24^, ^25^ The expression of alpha‐fetoprotein is regulated by PIK3R3 through the NF‐κB signaling pathway.^26^ However, few studies are available on the downstream molecules of PIK3R3, and the specific molecular mechanism of PIK3R3 in the growth of HCC has not been clarified. Further work is paracancerous needed to explore the downstream molecules of PIK3R3 and their role in the proliferation of HCC cells.

In this study, we further discovered that PIK3R3 activated Akt signaling to regulate downstream CDKN1C and SMC1A, promoting the proliferation of HCC cells. Our study sheds light on a new mechanism for HCC cell proliferation, which may aid in diagnosis and treatment.

## 2 MATERIALS AND METHODS

### 2.1 Agents, cell lines, and human samples

MK‐2206 2HCl was purchased from Selleck. MHCC97H and Hep3B cell lines were purchased from ZSBIO, China, and genotyping by STR was also performed by this company. The cells were cultured in DMEM supplemented with 10% fetal bovine serum and 1% penicillin–streptomycin. The cells were cultured in a humidified incubator at 37°C and 5% CO_2_.

This study was authorized by the Ethics Committee of Taizhou Central Hospital (Taizhou University Hospital) and was carried out according to the approved guidelines. Written consent was obtained when using the patient's surgical specimen.

The cancerous tissues and paracancerous tissues of 22 patients with diagnosed liver cancer were collected after surgical resection and were preserved at −80°C.

### 2.2 Gene expression analysis by RNA sequencing (RNA‐Seq)

The quality of the extracted total RNA was checked for purity, concentration, and integrity. The initial amount of total RNA was 1 μg for subsequent library construction. Novogene China was responsible for all library construction and sequencing work.

### 2.3 Quantitative real‐time PCR (qRT‐PCR)

TRIzol was used to extract the total RNA in each group, and RNA concentration was measured by NanoDrop. About 1000 ng of RNA was extracted from each group and reverse transcribed into cDNA by using the Thermo Scientific Maxima H Minus kit (Thermofisher). After mixing primer and 2X Universal SYBR Green Fast qPCR Mix (Abclonal), LightCycle 96 (Roche) was used for qPCR. All primers were purchased from Sangon, and the expression levels were normalized by quantitative analysis of the GAPDH gene. Primer sequences used to amplify the gene encoding PIK3R3 were as follows: 5′‐ TTTCCCAGGATGTTCAAGTGTA‐ 3′ and 5′‐ TTTGGAACTGCTGAAGTCATTG‐ 3′; For CDKN1C are 5′‐ ATCCACGATGGAGCGTCTTG‐3′ and 5′‐ TCGTAATCCCAGCGGTTCTG‐3′; For SMC1A are 5’‐GCTGGAGAAATTCAATCGAGAC‐3′ and 5′‐ TGCTAGTGGTGATGTATTCCTC‐3′. All experiments were performed in triplicate.

### 2.4 Western blot (WB)

Cells were collected in RIPA buffer (Thermofisher) containing protease inhibitors and phosphatase inhibitors (Thermofisher) for protein extraction. Protein concentration was determined using a BCA protein assay (Beyotime). The total lysate (50 μg each) was mixed with 5× SDS loaded buffer, heated at 95°C for 10 min, separated on 12% or 10% SDS−polyacrylamide gel, transferred to a 0.2 μm nitrocellulose membrane, and incubated in 5% bovine serum albumin at room temperature for 1 h. The following antibodies were used for WB analysis: PIK3R3 (Abclonal), GAPDH (Abclonal), AKT (CST), p‐AKT (CST), CDKN1C (Proteintech), and SMC1A (Proteintech).

### 2.5 Colony formation and 5‐Ethynyl‐2‐Deoxyuridine (EdU) assay

Cells in each group including PIK3R3 knockdown, PIK3R3 overexpression, CDKN1C+PIK3R3 knockdown, SMC1A overexpression+PIK3R3 knockdown, and control groups were prepared into single‐cell suspension and plated onto a six‐well plate at the density of 5000 cells per well. Colony formation was monitored continuously at 1, 5, and 10 days after clone establishment was confirmed at 6 h. Clone size was measured using an inverted microscope (ZEN2). For the EdU assay, cells undergoing DNA replication were detected using BeyoClick™ Edu‐594 Kit (Beyotime) according to the manufacturer's instructions.

### 2.6 Immunohistochemistry (IHC)

The paraffin sections of HCC tissue (*n* = 22) and paracancerous tissue (*n* = 22) were immunostained with PIK3R3, CDKN1C, and SMC1A antibodies. Preprocessing with dual endogenous enzyme blocker (Zsbio) for 30 min before staining. The primary antibody was incubated overnight at 4°C. After washing, the tissues were added with the appropriate amount of secondary antibody and incubated at 37°C for 30 min, followed by dropping the appropriate amount of DAB solution and restaining with hematoxylin. Neutral gum was added to cover the slides, and the slides were sealed. The staining results were examined by an inverted microscope (ZEN2).

### 2.7 Flow cytometry assay

Cell cycle distribution was determined by flow cytometry using PI staining Kit (Glenview) according to the manufacturer's guidelines. Cells with various treatments were harvested, washed three times with PBS, and fixed with 70% alcohol for 3 h at 4°C without light. The cell solution was centrifuged, and the supernatant was discarded. The residue was stained by PI solution for 30 min. Cells were harvested by centrifugation, and the percentage of each cycle was determined by BD FACS ARIA II flow cytometry (Becton Dickinson).

### 2.8 SiRNA, plasmid, and lentivirus transfection

For siRNA and plasmid transfection, the cells were seeded in six‐well plates or 6 cm dish and cultured for 24 h. Transfection system containing transfection reagent Lipofectamine™ RNAiMAX or Lipofectamine™ 2000 (Thermofisher), siRNA or plasmid, and optimum medium (Thermofisher) were prepared in 1.5 mL centrifuge tube and cultured for 30 min. The cells were added to the cocktail and mixed well by shaking. At 48 h after transfection, the cells were ready for use.

Lentiviral PIK3R3 and control vector were purchased from Genechem, Tec, and the sequences were copied from NM_001114172.1. MHCC97H and Hep3B cells were transfected by lentiviral particles at a multiplicity of infection of 20 followed by adding puromycin (2 μg/mL). The overexpression efficiency of PIK3R3 was evaluated by qRT‐PCR and WB analyses after the selection of a single clone.

### 2.9 Subcutaneous xenograft model

SPF‐grade Balb/c nude mice were provided by the Experimental Animal Center of the Second Xiangya Hospital, Central South University. All animal experiments followed the ethical principles of animal experiments and were carried out in strict accordance with operating standards. MHCC97H cells were transfected with PIK3R3 overexpression or empty lentivirus, and stable transfection cells were established. About 5 × 10^6^ cells were subcutaneously injected into the backside of mice. Tumor volume was measured every 5 days, and mice were sacrificed on Day 30. Tumor samples were collected for further examination.

### 2.10 Immunoprecipitation

To test protein interactions, immunoprecipitation was performed. MHCC97H cells were cultured and disrupted using sonication in NP‐40 lysis buffer with added cocktail and PhosSTOP cocktail tablets. The supernatants were incubated with specific antibodies overnight, followed by incubation with protein A/G beads on a rotating platform at 4°C for 4 h. The resulting immunoprecipitates were analyzed via western blotting after eliminating nonspecific adsorption using agarose beads.

### 2.11 Statistical analysis

Statistical analyses were performed with GraphPad software version 6.0. *T*‐test to assess significant differences between the two groups. For multiple‐group data, one‐way ANOVA was used. Values with a *p‐*value less than 0.05 were considered statistically significant.

## 3 RESULTS

### 3.1 PIK3R3 is upregulated in HCC and correlated with clinicopathological characteristics and patient survival

**FIGURE 1:**
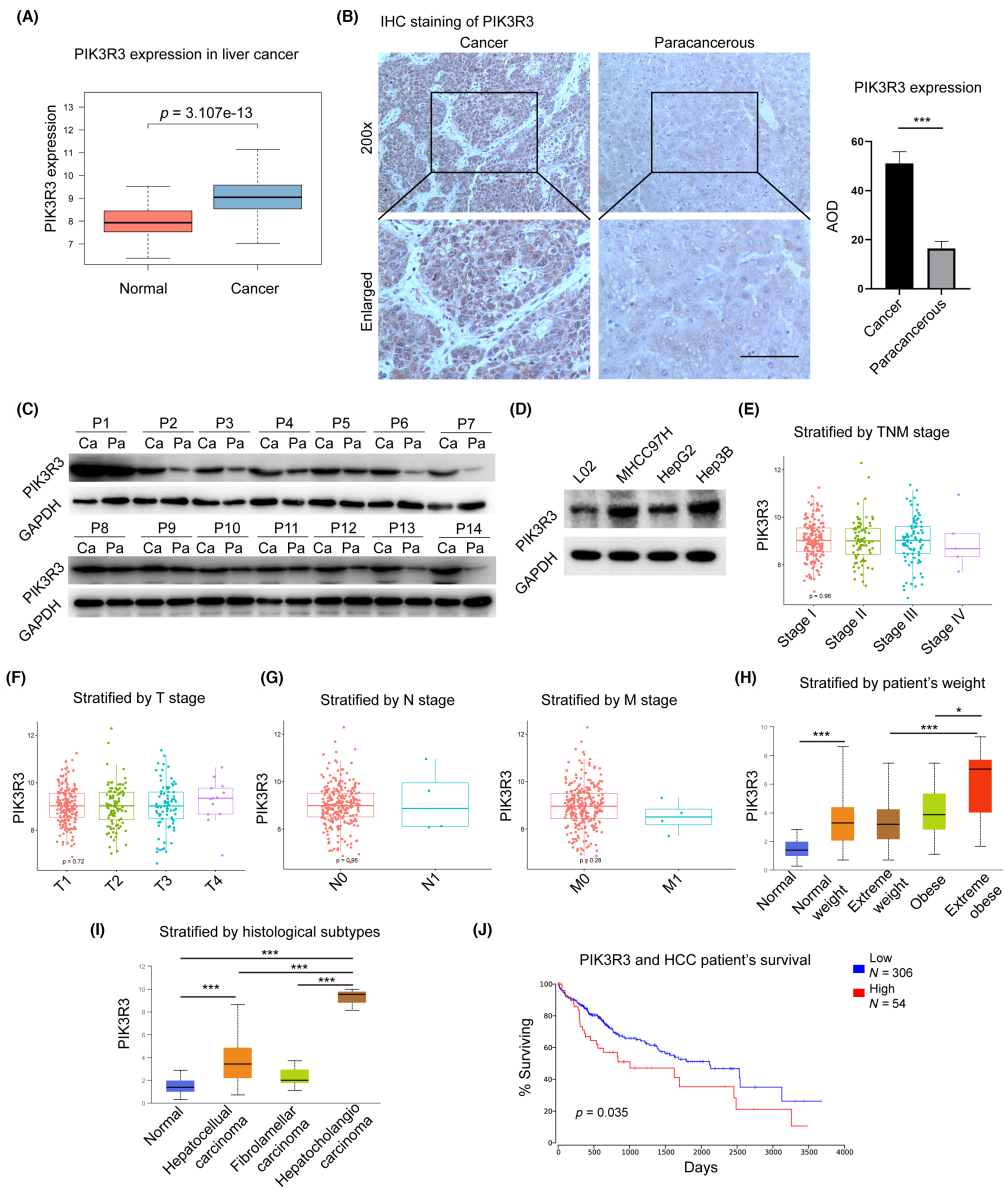
Phosphoinositide‐3‐kinase regulatory subunit 3 (PIK3R3) is upregulated in hepatocellular carcinoma (HCC) and is correlated with clinicopathological characteristics and patient survival. (A) The TCGA Liver cancer program was used to compare the expression of PIK3R3 in HCC, and the result showed that PIK3R3 was significantly upregulated in liver cancer compared with normal tissue. (B) Immunohistochemistry assay was used to explore the expression of the PIK3R3 protein in clinical samples. Compared with paracancerous tissues, the staining of PIK3R3 in cancer tissues was stronger. The bar indicates 100 μm. (C) Fresh samples from 14 patients were used to compare the protein expression of PIK3R3 between cancer and paracancerous tissues. (D) The expression levels of PIK3R3 in normal human liver cells (Lo2) and in MHCC97H, HepG2, and Hep3B cells. (E–G) Using TCGA clinic data, the expression of PIK3R3 was not significantly correlated with TNM, T, N, and M stages. (H) Expression of PIK3R3 was significantly correlated with the patient's weight, that is, the higher expression indicates more weight. (I) PIK3R3 is highly expressed in hepatocholangial carcinoma, hepatocellular carcinoma, and fibrolamellar carcinoma compared to normal liver tissue. (J) Using the Oncolnc online tool, higher expression of PIK3R3 indicated poor prognosis in patients with HCC. Ca, cancer; P, patient; Pa, paracancerous; *p < 0.05; ***p < 0.001.

To examine the expression of PIK3R3 in HCC and normal tissues, we retrieved the data from TCGA. A significant upregulation of PIK3R3 was observed in HCC compared with normal tissues (Figure 1A). The same result was obtained in the GSE database analysis (Figure S1A). Besides, PIK3R3 displayed a more pronounced variation in liver cancer compared to normal tissues, in contrast to PIK3R1 and PIK3R2 (Figure S1B–D).In fresh clinical samples, we confirmed that PIK3R3 was overwhelmingly upregulated in HCC compared with paired normal tissues (Figure 1B,C). The expression levels of PIK3R3 are higher in MHCC97H, HepG2, and Hep3B cells compared to normal human liver cells (Lo2) (Figure 1D).To determine the clinical significance of PIK3R3 in HCC, we correlated its expression with clinical parameters. Neither the expression of PIK3R3 RNA nor protein was significantly correlated with the T, M, N, or TNM stage of HCC (Figure 1E–G). Interestingly, the expression of PIK3R3 was associated with the patient's weight, that is, the higher expression indicates more weight (Figure 1H). PIK3R3 was elevated in hepatocholangial carcinoma compared with hepatocellular and fibrolamellar carcinoma (Figure 1I). To explore the prognostic value of PI3KR3 in HCC, we extracted data from the TCGA HCC program and reanalyzed using the Oncolnc online tool (www.oncolnc.org). High expression of PIK3R3 indicated poor survival for patients (Figure 1J). These data demonstrated that HCC upregulated the expression of PIK3R3, which was correlated with the patient's weight, histological subtype, and prognosis.

### 3.2 PIK3R3 controls HCC cell growth in vitro

**FIGURE 2:**
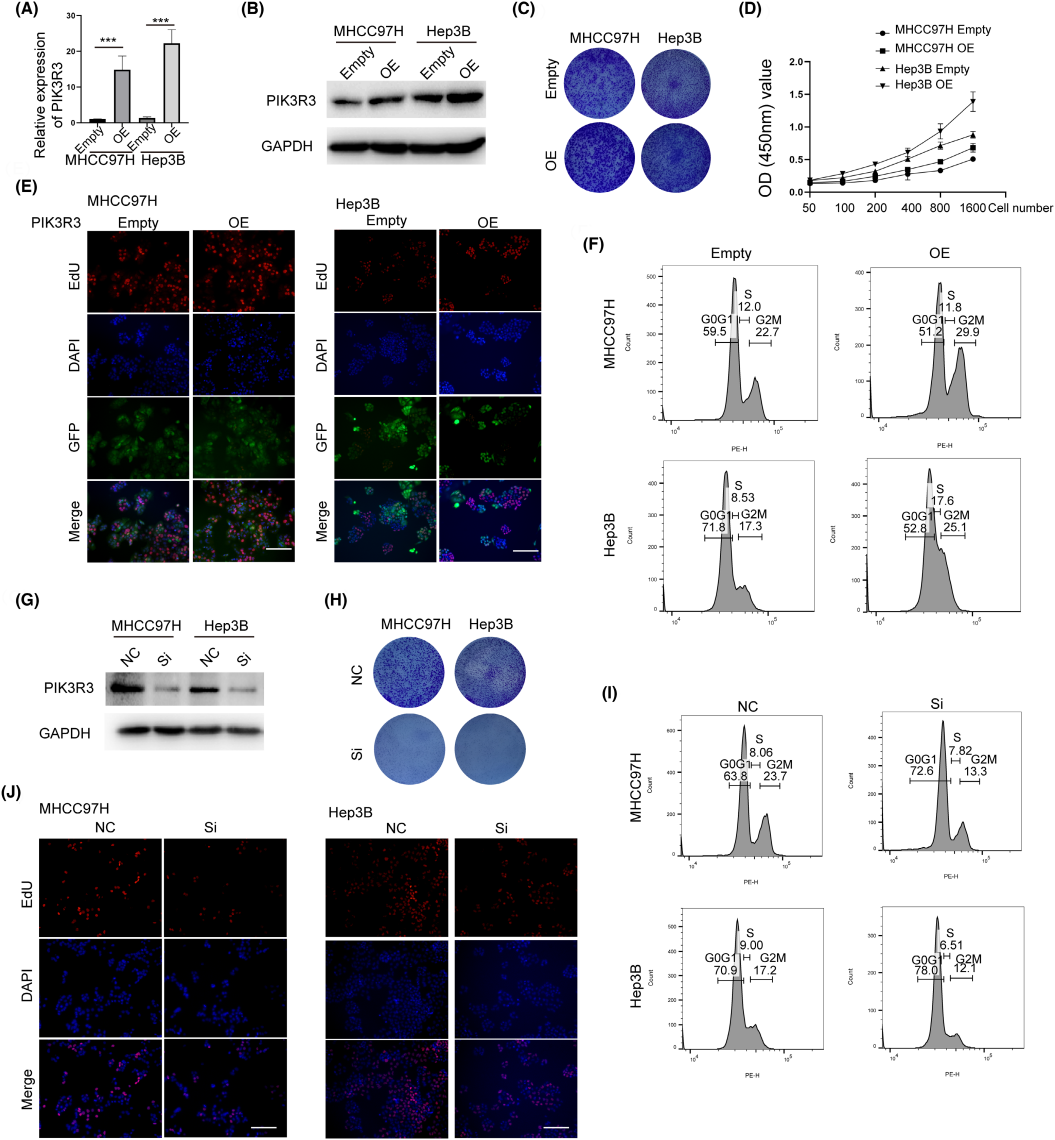
Phosphoinositide‐3‐kinase regulatory subunit 3 (PIK3R3) controls hepatocellular carcinoma (HCC) cell growth in vitro. (A) PIK3R3 was cloned into a plasmid and carried by lentivirus and transfected by MHCC97 and Hep3B cells. quantitative real‐time‐PCR confirmed that PIK3R3 was significantly upregulated by lentivirus in both cell lines. (B) Western blot (WB) showed that PIK3R3 was upregulated by lentivirus in both cell lines. (C) Colony formation assay showed that the overexpression of PIK3R3 increased the size of the colony formed compared with the empty in MHCC97 and Hep3B cells. (D) CCK‐8 assay was used to determine the growth rate of HCC cells upon PIK3R3 overexpression and showed that PIK3R3 overexpression significantly improved the growth rate of HCC cells. The bar indicates 100 μm. (E) 5‐Ethynyl‐2‐Deoxyuridine (EdU) assay showed that overexpression of PIK3R3 improved cell growth in HCC cells. (F) Cell cycle distribution showed that the obvious increased G2M was induced by PIK3R3 overexpression in HCC cells. (G) siRNA was used to knock down PIK3R3 in MHCC97H and Hep3B cells. WB showed that siRNA successfully knocked it down in both cells. (H) Knockdown of PIK3R3 impaired the colony formation of HCC cells. (J) EdU assay showed that the knockdown of PIK3R3 decreased cell growth in vitro. The bar indicates 100 μm. (I) Cell cycle distribution analyzed by flow cytometry showed an obvious decreasing trend of G2M in HCC cells. NC, negative control; OE, overexpression; Si, siRNA; ***p < 0.001.

Two cell lines were selected to explore the underlying function of PIK3R3 in HCC cells. PIK3R3 was cloned in a plasmid equipped with the GFP gene and packaged in lentivirus. Stably transfected cells were obtained followed by the manufacturer's guidelines. qRT‐PCR and WB analyses demonstrated that PIK3R3 was successfully overexpressed in MHCC97H and Hep3B cells (Figure 2A,B). Colony formation assays showed that overexpression of PIK3R3 promoted cell growth compared with the control group in both cell lines (Figure 2C). Quantitatively, the cell growth rate was significantly improved upon PIK3R3 overexpression (Figure 2D). EdU assay was also used, and the result showed the same trend in both cell lines (Figure 2E). Cell cycle distribution was determined by flow cytometry and showed that overexpression of PIK3R3 improved G2M distribution compared with the control group (Figure 2F). Furthermore, we used siRNA to knock down PIK3R3 in HCC cells. An optimal siRNA was selected by verification of protein expression (Figure 2G). As predicted, the knockdown of PIK3R3 significantly decreased the cell growth rate, impaired the ability of colony formation, and blocked the cell cycle in the G2M stage in MHCC97H and Hep3B cells (Figure 2H–J). Transfection of PIK3R3‐siRNA was performed, and after digestion of the cells, they were replanted in a six‐well plate. The following day, we observed that there were more floating cells, indicating that knocking down PIK3R3 did indeed affect cell replanting. However, we did not conduct further research on this (data not shown). These data firmly demonstrated that PIK3R3 controlled HCC cell growth in vitro.

### 3.3 PIK3R3 controls HCC cell growth in vivo

**FIGURE 3:**
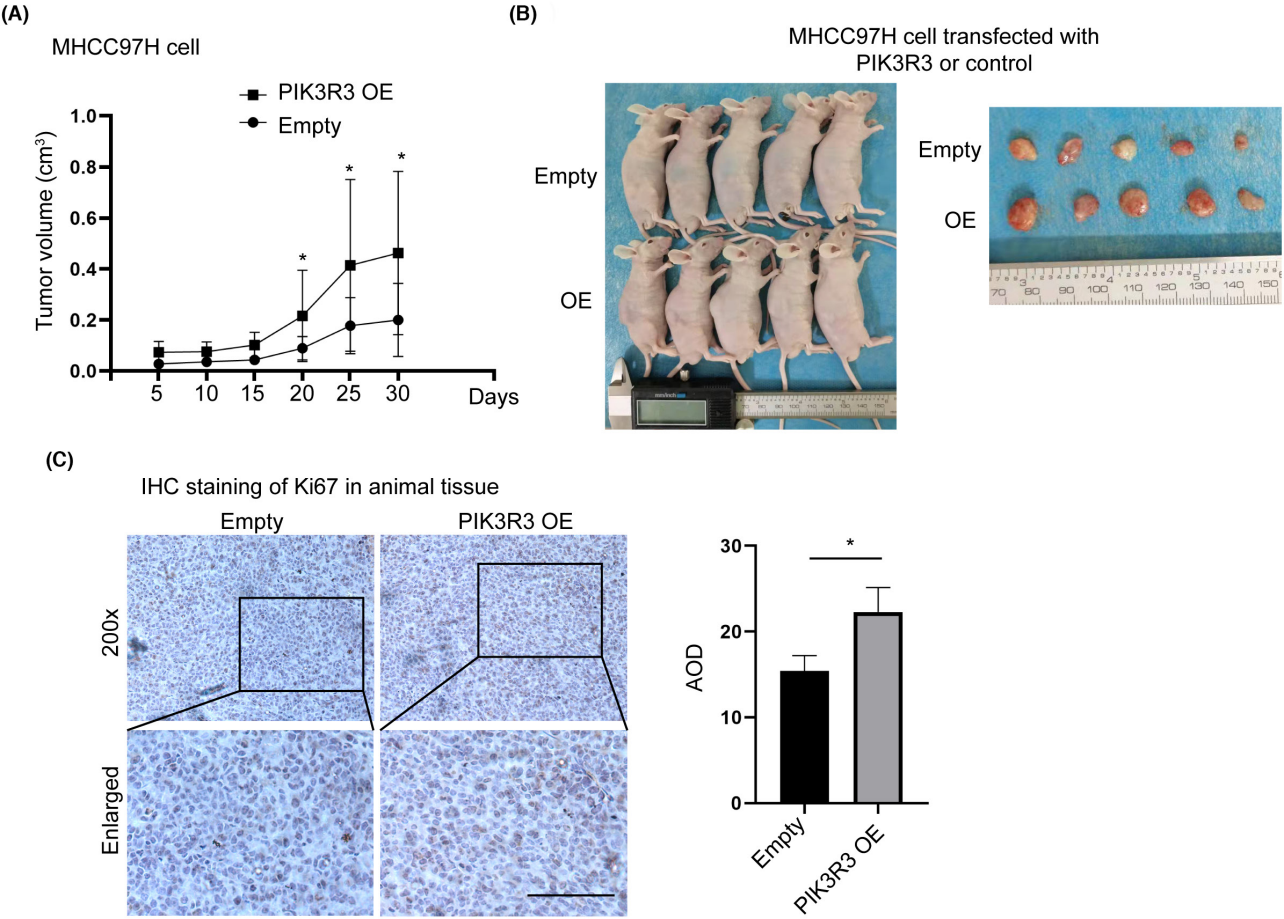
Phosphoinositide‐3‐kinase regulatory subunit 3 (PIK3R3) controls hepatocellular carcinoma cell growth in vivo. (A) MHCC97 cells transfected with PIK3R3, or empty were subcutaneously injected into mice. Tumor volume was examined every 5 days, and the mice were sacrificed on Day 30. The tumor volume of the PIK3R3 overexpression group was significantly larger than that of the control group at Days 20, 25, and 30. (B) The tumor was cut out and displayed by the group. Overexpression of PIK3R3 improved the tumor size compared with the empty group. (C) Tumor tissue was analyzed by immunohistochemistry staining and showed that the overexpression of PIK3R3 improved the positive rate of Ki67 staining compared with the empty group. OE, overexpression; *p < 0.05.

We then examined the effect of PIK3R3 in vivo. MHCC97H cells with stable transfection of PIK3R3 and control were subcutaneously injected into BALB/c nude mice. Tumor volume was recorded every 5 days and showed that overexpression of PI3KR3 significantly improved the tumor volume compared with the control (Figure 3A). Tumor tissue was collected, and the tumor size of the PIK3R3 overexpression group was larger than the control (Figure 3B). Ki67 staining confirmed that the overexpression of PIK3R3 promoted HCC cell growth in vivo (Figure 3C). These data demonstrated that overexpression of PIK3R3 promoted HCC growth in vivo.

### 3.4 Dysregulation of CDKN1C and SMC1A is responsible for PIK3R3 knockdown‐mediated effect on HCC

**FIGURE 4:**
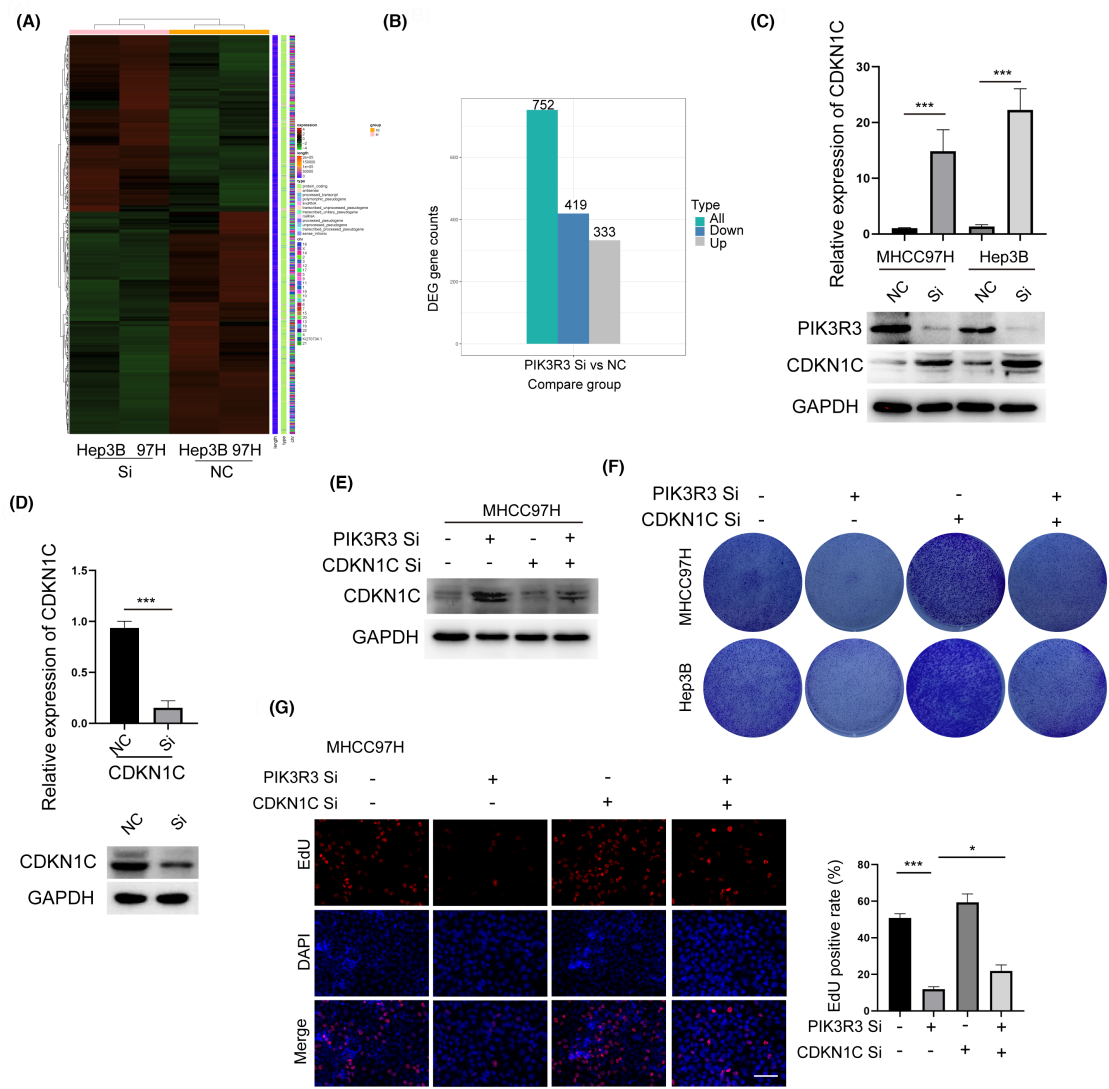
Upregulation of CDKN1C is partially responsible for phosphoinositide‐3‐kinase regulatory subunit 3 (PIK3R3) knockdown‐mediated effect in hepatocellular carcinoma. (A) Global gene expression variations were determined by RNA sequence in MHCC97H and Hep3B cells upon PIK3R3 knockdown. The heatmap showed the overall expression of mRNA in both cell lines. (B) Using the cut‐off value of the absolute value of log_2_ fold change = 1 and *p* < 0.05, 752 differentially expressed genes were screened. Among them, 419 genes were dysregulated upon PIK3R3 knockdown, and 333 genes were upregulated in MHCC97H and Hep3B cells. (C) Consistent with the result of RNA sequencing, Western blot showed that CDKN1C was upregulated when PIK3R3 was knocked down by siRNA in cell lines. (D) CDKN1C was knocked down by siRNA, and the efficiency was confirmed at RNA and protein levels in MHCC97H. (E) CDKN1C siRNA largely eliminated the effect of PIK3R3 knockdown upon the expression of CDKN1C. (F) As shown by the colony formation assay, CDKN1C siRNA increased tumor growth in vitro and largely rescued the effect of PIK3R3 knockdown in MHCC97H and Hep3B cells. (G) A similar result was observed by 5‐Ethynyl‐2‐Deoxyuridine (EdU) assay, that is, CDKN1C knocked down significantly and rescued the inhibition effect of PIK3R3 knockdown on cell growth. NC, negative control; Si, siRNA; *p < 0.05; ***p < 0.001.

Global gene expression variations were determined by RNA sequence in PIK3R3 knockdown and control cells to explore the mechanism of PIK3R3‐mediated cell growth in HCC (Figure 4A). Using the cut‐off value of the absolute value of log_2_ fold change = 1 and *p* < 0.05, 419 genes were dysregulated between PIK3R3 knockdown and control groups in MHCC97H and Hep3B cells (Figure 4B). As our aforementioned data indicated the strong growth control ability of PIK3R3, we focused on genes associated with tumor growth. Our results indicated a higher correlation between CDKN1C and PIK3R3 compared to CDKN1A and CDKN1B (Figure S2A,B). CDKN1C is a cyclin‐dependent kinase inhibitor playing an essential role in HCC. Knockdown of PIK3R3 significantly increased the RNA and protein expression of CDKN1C in MHCC97H and Hep3B cells (Figure 4C). We used CDKN1C siRNA to knock down it in MHCC97H cells, and we selected the most efficient one for the following experiments (Figure 4D,E). Expectedly, CDKN1C siRNA could rescue the effect of PIK3R3 knockdown that the ability of colony formation was largely recovered (Figure 4F). The EdU assay showed the same trend in both cell lines (Figure 4G). These data clearly showed that the upregulation of CDKN1C was partially responsible for PIK3R3 knockdown‐mediated effect on HCC.

**FIGURE 5:**
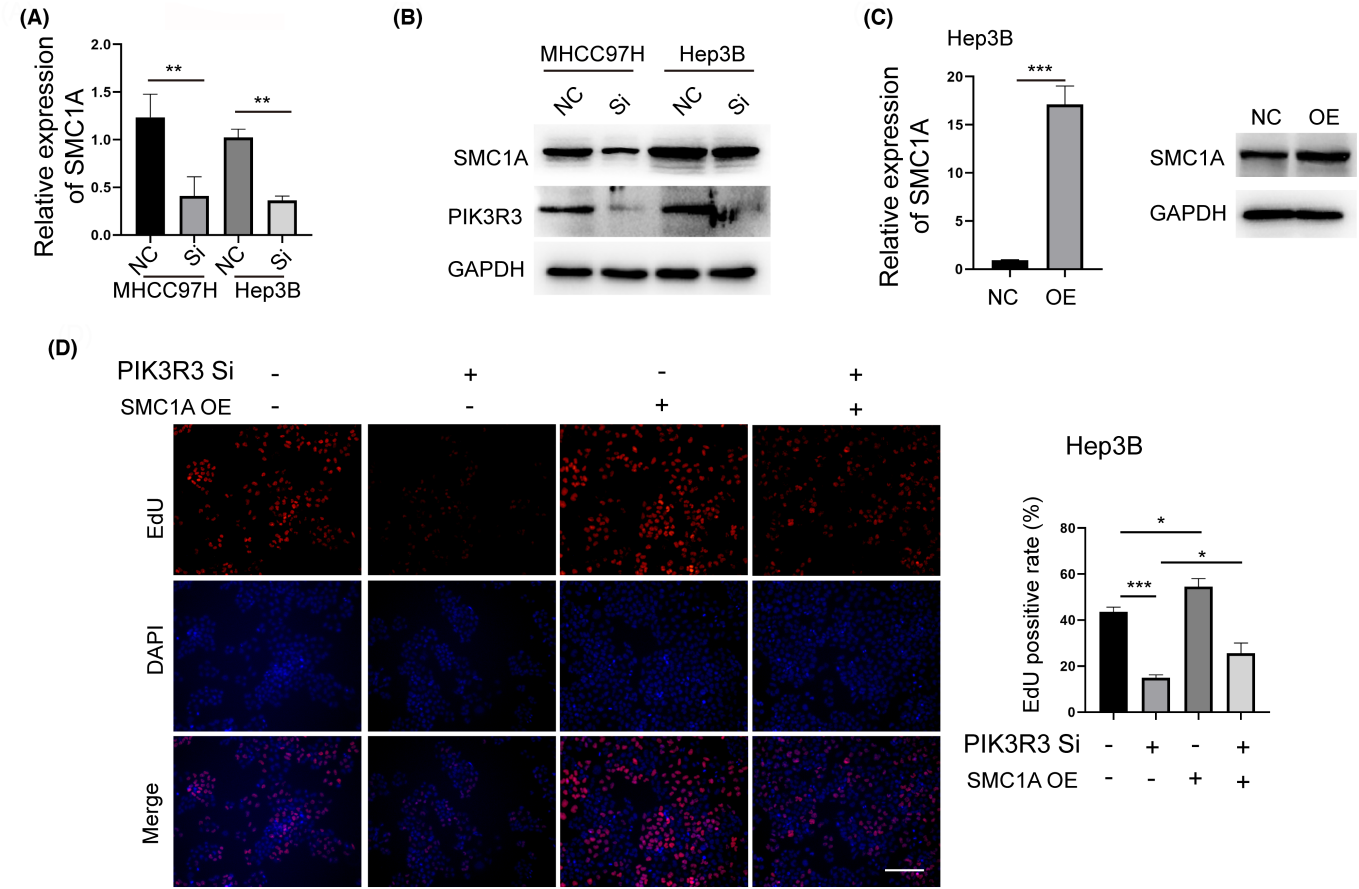
Downregulation of SMC1A is partially responsible for phosphoinositide‐3‐kinase regulatory subunit 3 (PIK3R3) knockdown‐mediated effect on hepatocellular carcinoma. (A) The result of quantitative real‐time (qRT)‐PCR confirmed that PIK3R3 knockdown significantly decreased the expression of SMC1A in MHCC97H and Hep3B cells. (B) SMC1A protein showed a consistent trend with the expression of PIK3R3 in both cell lines. (C) SMC1A was upregulated by SMC1A plasmid and confirmed by qRT‐PCR and Western blot assay in Hep3B cells. (D) Overexpression of SMC1A significantly rescued the inhibition effect of PIK3R3 knockdown on Hep3B cells. NC, negative control; OE, overexpression; Si, siRNA; *p < 0.05; ***p < 0.001.

In addition, we observed that SMC1A was significantly downregulated upon PIK3R3 knockdown in MHCC97H and Hep3B cells (Figure 5A,B). SMC1A is a member of the SMC superfamily and a core component of the cohesin complex essential for sister chromatid cohesion and plays an essential role in cancer. We used SMC1A plasmid to overexpress it in MHCC97H and Hep3B cells (Figure 5C). Consistent with previous work, the overexpression of SMC1A improved HCC cell growth in vitro illustrated by the EdU assay (Figure 5D). Furthermore, SMC1A largely rescued the effect of PIK3R3 knockdown on MHCC97H and Hep3B cells (Figure 5D). Thus, these data collectively showed that the dysregulation of CDKN1C and SMC1A is responsible for PIK3R3 knockdown‐mediated effect on HCC cells. While the Immunoprecipitation experiment demonstrated there is indirect interaction between PIK3R3 and CNKN1C or SMC1A. (Figure S2C).

### 3.5 Expression of PIK3R3 is intimately associated with CDKN1C and SMC1A in clinical and animal samples

**FIGURE 6:**
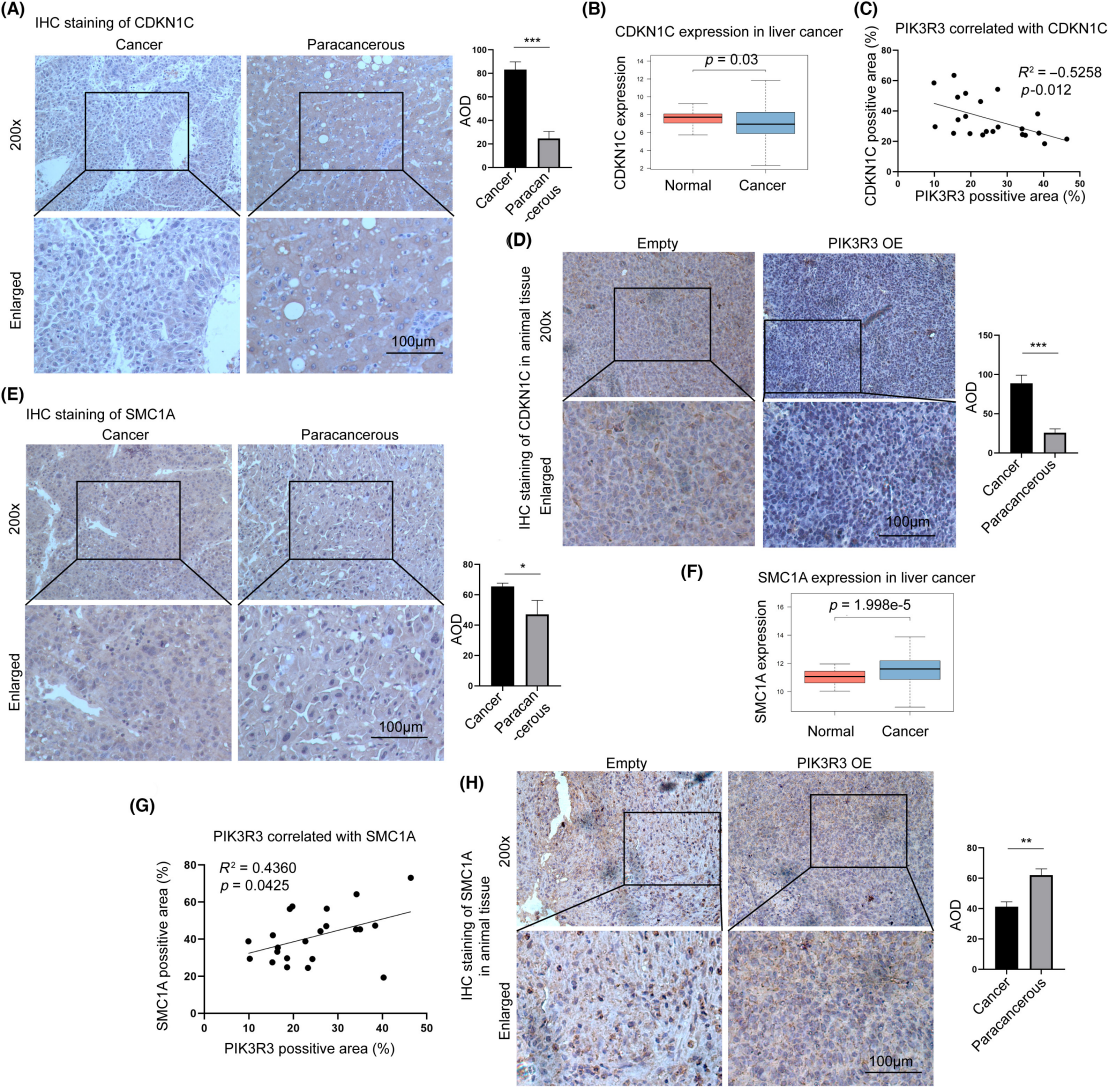
Phosphoinositide‐3‐kinase regulatory subunit 3 (PIK3R3) is intimately associated with CDKN1C and SMC1A in clinical and animal samples. (A) Immunohistochemistry (IHC) staining of CDKN1C in clinical samples showed that CDKN1C was downregulated in cancer tissue compared with the paired precancer tissue. (B) Data from the TCGA hepatocellular carcinoma (HCC) program confirmed the result of clinical samples. (C) The expression of CDKN1C and PIK3R3 was examined by IHC staining in 22 clinical samples, and the correlation analysis between them was determined. A clear negative correlation was found between CDKN1C and PIK3R3 in HCC samples. (D) In animal tissue, PIK3R3 overexpression inhibited the expression of CDKN1C. (E) IHC staining of SMC1A in clinical samples showed that SMC1A was downregulated in cancer tissue compared with that in the paired paracancerous tissue. (F) Data from the TCGA HCC program confirmed the result of clinical samples. (G) A positive correlation was found between PIK3R3 and SMC1A in clinical samples. (H) Overexpression of PIK3R3 increased the staining of SMC1A in animal samples compared with empty groups. OE, overexpression; The IHC staining intensity was represented by the positive area in a slide.

To correlate the expression of PIK3R3 with CDKN1C and SMC1C in vivo, we examined the expression of these genes in clinical and animal samples. Consistent with the previous works, CDKN1C was significantly downregulated in HCC compared with normal tissues, and the data from TCGA further confirmed it (Figure 6A,B). A significant correlation between PIK3R3 and CDKN1C was observed in clinical samples (Figure 6C). Higher expression of PIK3R3 indicated lower expression of CDKN1C in animal models (Figure 6D). In addition, the TCGA data and our clinical samples showed significant overexpression of SMC1A in HCC compared with the control (Figure 6E,F). The relationship between PIK3R3 and SMC1A was also examined, and a significant correlation between them was found in clinical samples (Figure 6G). Compared with the control, the overexpression of PIK3R3 improved the expression of SMC1A in animal models (Figure 6H). These data demonstrated that PIK3R3 was intimately associated with CDKN1C and SMC1A in clinical and animal samples.

### 3.6 PIK3R3/Akt signaling regulates the expression of CDKN1C and SMC1A in HCC cells

**FIGURE 7:**
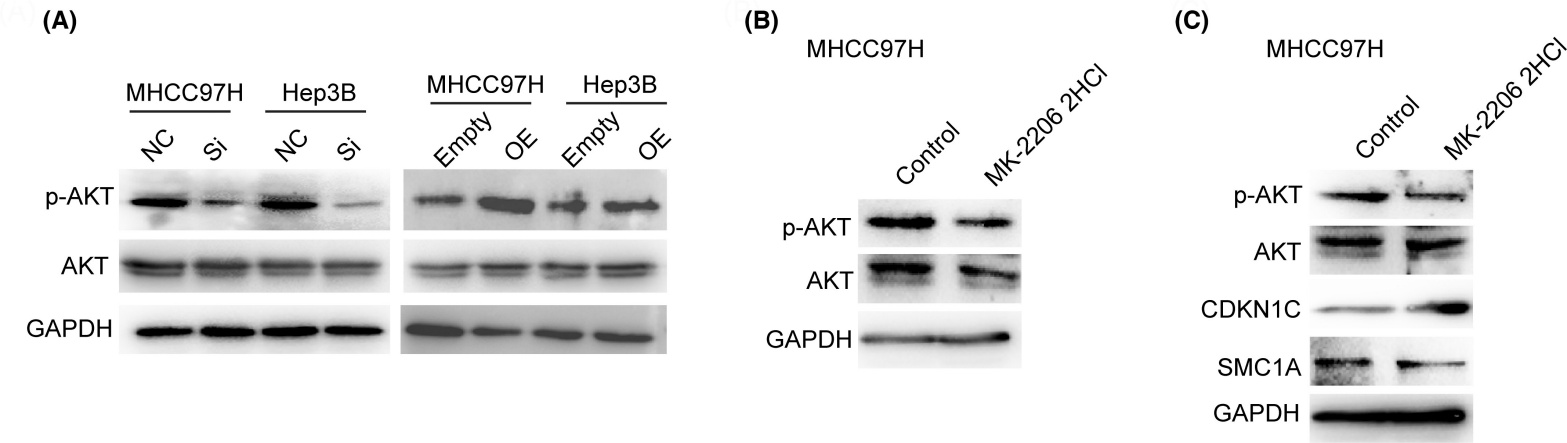
Phosphoinositide‐3‐kinase regulatory subunit 3 (PIK3R3)/Akt signaling regulates CDKN1C and SMC1A in hepatocellular carcinoma cells. (A) Western blot showed that p‐AKT was inhibited by PIK3R3 knocked down but activated by PIK3R3 overexpression in MHCC97H and Hep3B cells. (B) MK‐2206 2HCl inhibited the activation of p‐AKT in MHCC97H cells. (C) Inhibition of p‐AKT by MK‐2206 2HCl increased the expression of CDKN1C but reduced the expression of SMC1A, similar to the result of PIK3R3 knockdown. NC, negative control; OE, overexpression; Si, siRNA.

As PIK3R3 is a member of the PI3K family, the aberrant activation of PI3K signaling is a common oncogenic event and triggers the activation of Akt in cancer. We found that knockdown of PIK3R3 could deactivate Akt in MHCC97H and Hep3B cells, but overexpression led to obvious activation of Akt (Figure 7A). MK‐2206 2HCl is a highly specific inhibitor of Akt. The effect of Akt activation by overexpression of PIK3R3 was almost attenuated by MK‐2206 2HCl in HCC cells (Figure 7B). Interestingly, the administration of MK‐2206 2HCl increased the expression of CDKN1C but decreased the expression of SMC1A in HCC cells (Figure 7C). Thus, PIK3R3 activated Akt signaling in HCC cells and determined the expression of CDKN1C and SMC1A, two downstream of PIK3R3. Further exploration showed the direct mechanism of PIK3R/Akt signaling on the regulation of CDKN1C and SMC1A in HCC cells.

## 4 DISCUSSION

HCC is one of the most common malignant tumors of the digestive system and has high mortality and increasing incidence. The tumor size of HCC determines the treatment plan to a certain extent. Surgical liver resection, local ablation, and liver transplantation are promising for radical treatments of HCC. However, most clinical guidelines recommend these strategies to be appropriate only for the early stage of HCC.^26^ Therefore, early detection and treatment of patients with HCC can achieve a better prognosis. Specific molecular mechanisms related to the growth of HCC should be explored to provide new targets for HCC treatments.

PIK3R3, also called p55PIK, is overexpressed in a variety of tumors and is associated with tumor development, metastasis, and prognosis. It is believed to have oncogene functions. For example, PIK3R3 expression has been upregulated in HCC, colorectal cancer, cervical cancer, and non‐small cell lung cancer.^18^, ^20^, ^21^, ^25^ A study showed that EVA1 could promote the growth and metastasis of HCC by regulating the ERBB signaling transduction complex including PIK3R3.^25^ However, most studies have explored the mechanism of upstream molecules of PIK3R3, especially the negative regulation of microRNA by targeting PIK3R3. The direct downstream molecules and specific mechanisms of PIK3R3 in HCC are still unclear. This study aims to explore the mechanism of PIK3R3 on the proliferation of HCC cells and the downstream molecules of PIK3R3. We found that PIK3R3 upregulated the expression of CDKN1C, downregulated the expression of SMC1A, and activated Akt signaling, thereby promoting HCC cell proliferation and tumor growth.

First, we confirmed the upregulation of PIK3R3 in HCC by using the TCGA database and further verified it in clinical and animal samples. We found that the overexpression and knockdown of PIK3R3 in HCC cells affected their proliferation function in vitro and the growth of subcutaneous transplanted tumors in vivo, suggesting that the function of PIK3R3 is related to the cell proliferation of HCC. Consistent with previous studies, the overexpression of PIK3R3 has been reported to benefit cell proliferation of HCC, colorectal cancer, and oral squamous cell carcinoma, and vice versa.^21^, ^22^, ^27^ Therefore, we focused on the specific mechanism of the effect of PIK3R3 on the proliferation of HCC cells and discovered the dysregulated genes in HCC cells with PIK3R3 knockdown by RNA sequence. Among 419 dysregulated genes, CDKN1C and SMC1A, which are closely related to the cell cycle, attracted our attention, and their expression levels significantly increased and decreased after PIK3R3 knockdown, respectively. We hypothesized that PIK3R3 regulates the proliferation ability by regulating the expression of CDKN1C and SMC1A and affecting the cell cycle of HCC. In the following experiments, we knocked down CDKN1C and overexpressed SMC1A to confirm the above hypothesis. The colony formation and cell growth capacity of HCC cells damaged by the knockdown of PIK3R3 were significantly restored. On the other hand, the co‐expression relationship between PIK3R3 and CDKN1C or SMC1A has been verified in clinical and animal samples as well as the TCGA database. Finally, we selected Akt signaling as the specific signaling pathway. A previous study showed that the Akt pathway promoted liver tumorigenicity by downregulation of CDKN1C.^28^ Another study showed that SMC1A knockdown significantly inhibited the cell proliferation and activation of Akt in colorectal cancer.^29^

## 5 CONCLUSION

In conclusion, knockdown PIK3R3 inhibits the tumor growth of HCC by upregulating the expression of CDKN1C and downregulating the expression of SMC1A by deactivating the Akt pathway and inhibiting cell proliferation and colony formation. Knockdown of CDKN1C or overexpression of SMC1A could relieve this inhibition. These results suggest that PIK3R3, CDKN1C, and SMC1A could be promising treatment strategies for HCC that deserve further investigation. However, the current study only proved that CDKN1C and SMC1A are downstream molecules of PIK3R3/Akt through functional loss and functional acquisition experiments. However, we need further research to clarify the specific mechanism of PIK3R3/Akt in regulating the expression of CDKN1C and SMC1A.

## AUTHOR CONTRIBUTIONS

**Weidong Lin:** Funding acquisition (supporting); investigation (supporting); project administration (supporting); supervision (supporting); writing – original draft (supporting); writing – review and editing (supporting). **Kunpeng Wang:** Conceptualization (equal); data curation (supporting); formal analysis (lead); funding acquisition (supporting); investigation (supporting); project administration (lead); writing – original draft (lead). **Jinggang Mo:** Data curation (supporting); methodology (supporting); resources (lead); supervision (supporting); visualization (lead). **Liezhi Wang:** Funding acquisition (supporting); project administration (supporting); software (supporting). **zhenshun Song:** Supervision (supporting); validation (supporting); visualization (supporting). **Hao Jiang:** Conceptualization (supporting); writing – original draft (supporting); writing – review and editing (supporting). **Cong Wang:** Conceptualization (supporting); data curation (supporting); supervision (supporting); writing – original draft (supporting). **Chong Jin:** Conceptualization (lead); funding acquisition (lead); investigation (lead); project administration (lead); supervision (supporting); visualization (supporting); writing – review and editing (lead).

## FUNDING INFORMATION

This project was supported by the grants from Zhejiang Province Public Welfare Technology Application Research Project (No. LGF21H160022), the Natural Science Foundation of Zhejiang Province (No. LQ22H160055), Science and Technology Plan Project of Taizhou (No. 21ywb26, 21ywb29, and 22ywa14), Medical Science and Technology Project of Zhejiang Province (No. 2017KY711, 2023KY403, 2023KY404, and 2023KY1340), and Project of Taizhou University (No.2018PY057).

## Supporting information


Figure S1.

Figure S2.

## Data Availability

We will provide original data when requested.
