# Supplementary figures and images for: PIK3R3 is upregulated in liver cancer and activates Akt signaling to control cancer growth by regulation of CDKN1C and SMC1A
Source: Cancer Med. 2023 May 22;12(13):14413–25. doi: 10.1002/cam4.6068 (PMC10358214; doi:10.1002/cam4.6068)

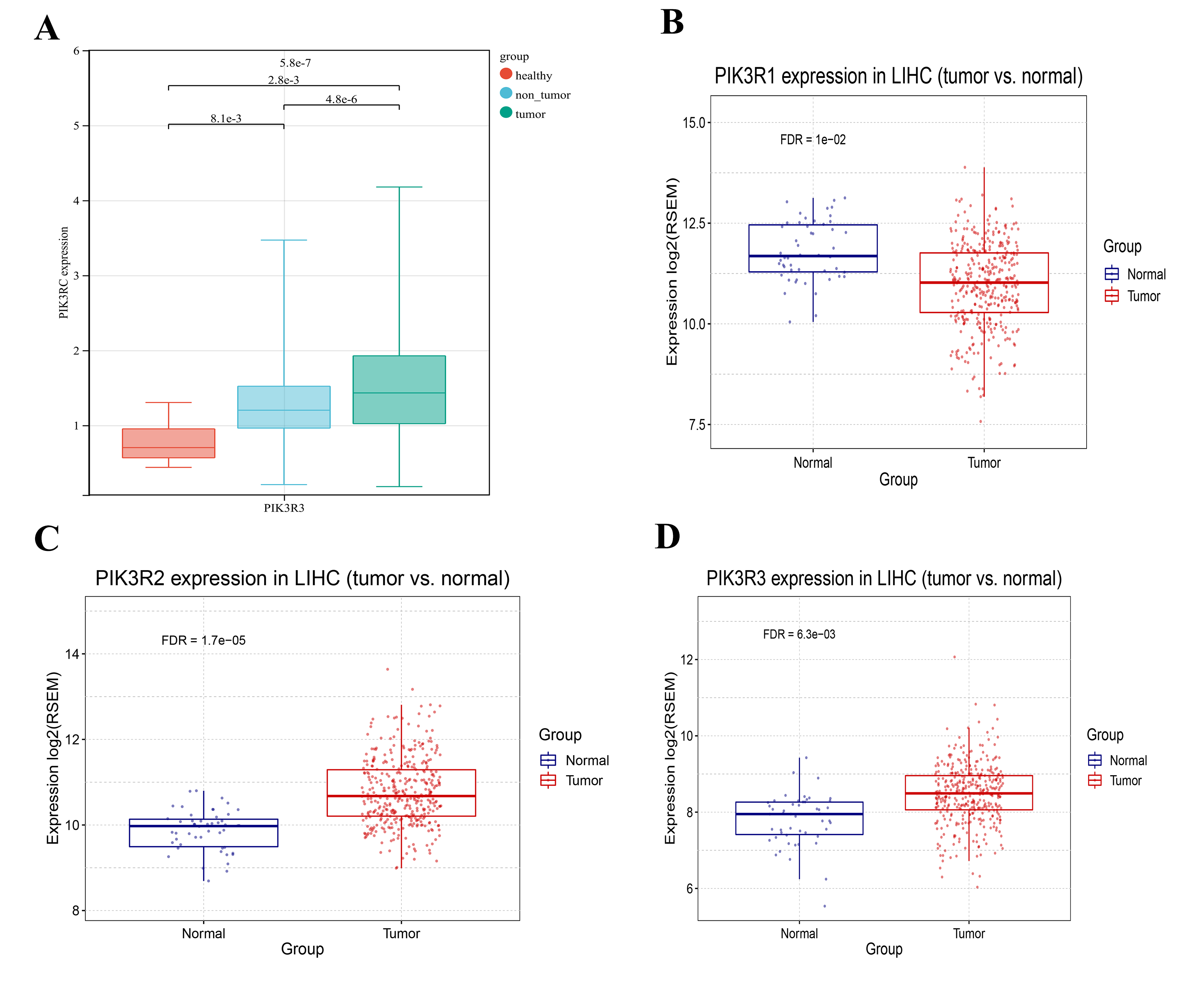

Supplement: Supplementary file 1 — Figure S1. [file CAM4-12-14413-s001.tif]

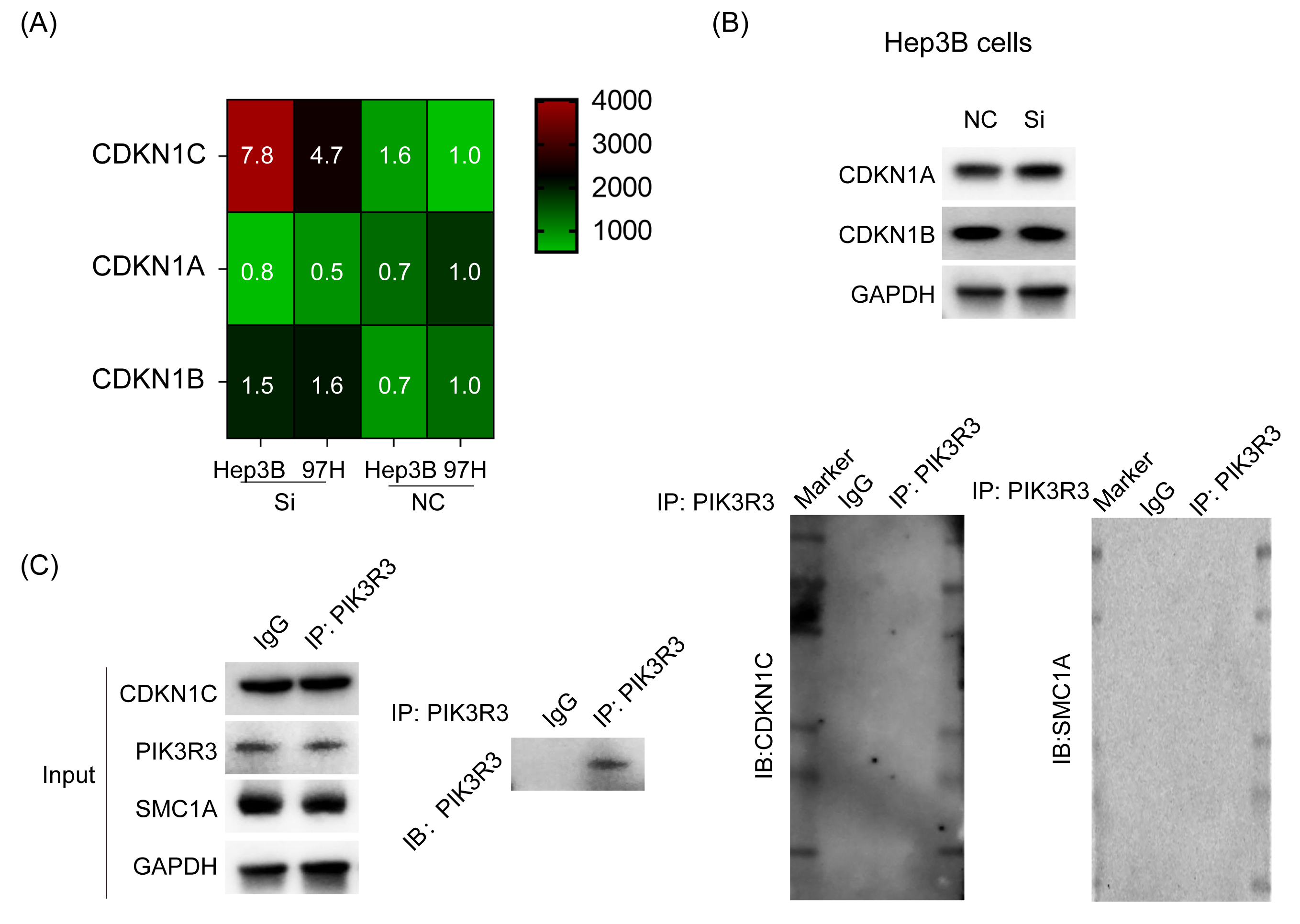

Supplement: Supplementary file 2 — Figure S2. [file CAM4-12-14413-s002.tif]
